# Screening and Characterization of an α-Amylase Inhibitor from *Carya cathayensis* Sarg. Peel

**DOI:** 10.3390/foods12244425

**Published:** 2023-12-10

**Authors:** Xiaosan Zhang, Guangrong Huang, Hua Liu, Wenwei Chen, Jing Zhao, Zhenbao Jia, Fei Tao

**Affiliations:** 1 Key Laboratory of Specialty Agri-Product Quality and Hazard Controlling Technology of Zhejiang Province, College of Life Sciences, China Jiliang University, Hangzhou 310018, China; zhangxsan33@163.com (X.Z.);; 2 Food and Drug Inspection and Testing Center of Chunan County, Hangzhou 310022, China; 3 College of Standardization, China Jiliang University, Hangzhou 310018, China

**Keywords:** *Carya cathayensis* Sarg. Peel, postprandial hyperglycemia, 5-*O*-*p*-coumaroylquinic acid, α-amylase inhibitor

## Abstract

Inhibiting α-amylase can lower postprandial blood glucose levels and delay glucose absorption, offering an effective approach for the development of antidiabetic diets. In this study, an active constituent with inhibitory activity against α-amylase was isolated and purified by bioassay-guided fractionation from *Carya cathayensis* Sarg. peel (CCSP). The active constituent was identified by NMR and Q-Exactive Orbitrap Mass Spectrometry as 5-*O-p*-coumaroylquinic acid (5-CQA). 5-CQA possessed strong inhibitory activity against α-amylase, with an IC50 value of 69.39 µM. In addition, the results of the kinetic study indicated that 5-CQA was a potent, reversible, noncompetitive inhibitor against α-amylase. The findings indicate that 5-CQA derived from CCSP has potential as a novel inhibitor against α-amylase, which can help mitigate postprandial blood sugar spikes, making it suitable for inclusion in antidiabetic diets.

## 1. Introduction

Starch is the primary carbohydrate storage molecule in grains and is a significant source of energy for the human body. Starch digestive enzymes, α-amylase and α-glucosidase, play pivotal roles in the digestive breakdown and subsequent absorption of dietary starches [1]. Specifically, α-amylase catalyzes the hydrolysis of the α-D-1,4-glucosidic bonds in starch, yielding dextrins and oligosaccharides [2]. Subsequently, α-glucosidase facilitates the conversion of these dextrins and oligosaccharides into glucose [3]. Glucose undergoes cellular respiration to produce ATP, which cells then utilize to perform various vital functions. Nonetheless, excessive consumption of starch, especially from refined sources, may heighten the risk of chronic conditions, including type 2 diabetes, cardiovascular disease, and specific cancers, attributable to the elevated glycemic index associated with these foods [4,5]. Targeting the digestive mechanisms of starch, specifically through the inhibition of starch hydrolases, offers a promising strategy for promoting foods with a reduced glycemic index [6]. Inhibitors against α-amylase are used as antidiabetic drugs to control postprandial hyperglycemia. However, these pharmaceuticals have been reported to cause side effects, such as diarrhea, flatulence, and stomachache [7]. Acarbose, a notable α-amylase inhibitor, impedes the breakdown of starch and sucrose, thereby moderating the absorption of starch from food [8]. However, its use is somewhat limited due to side effects like flatulence and other symptoms stemming from carbohydrate malabsorption [9]. In contrast, natural food sources such as vegetables, fruits, and nuts are abundant in phytochemicals, including potent secondary metabolites like flavonoids, phenolic acids, and saponins, which exhibit α-amylase inhibitory activity [10]. The extraction of α-amylase inhibitors from plant sources is increasingly regarded as sustainable, given the lower environmental impact of plant cultivation and harvesting compared to chemical synthesis, which often involves the use of hazardous substances and can generate significant waste [11]. Therefore, it is particularly necessary to screen for α-amylase inhibitors from natural plants.

Enzyme inhibitors are broadly classified into two main categories: reversible and irreversible [12]. Reversible inhibitors, which are the focus of extensive scientific research, can be further subdivided based on their effect on the Michaelis–Menten constant (K_m_) and the maximum velocity (V_max_) with increasing concentrations. These subcategories include competitive inhibitors, noncompetitive inhibitors, uncompetitive inhibitors, and mixed-type inhibitors [13]. In the realm of α-amylase inhibition, a variety of inhibitors have been identified from natural sources. Kawamura-Konishi et al. isolated a novel phlorotannin, 2-(4-(3,5-dihydroxyphenoxy)-3,5-dihydroxyphenoxy) benzene-1,3,5-triol (DDBT), from *Sargassum patens*, demonstrating competitive inhibition of α-amylase [14]. Yang et al. reported that silibinin, extracted from the seeds of *Silybum marianum*, functions as a noncompetitive inhibitor against α-amylase and exhibits synergistic inhibition when combined with acarbose [15]. Fan et al. purified a natural blue pigment from the leaves of *Vaccinium bracteatum* thunb. using a concise method. Their study indicated that this blue pigment acted as an uncompetitive inhibitor against α-amylase, with most of its inhibitory activity retained after in vitro simulated digestion [16]. Zhang et al. explored the bioactivity of chestnut inner skin extract (CISE). Their in vitro study revealed that CISE inhibited α-amylase as a mixed-type inhibitor. This activity led to a reduced rate of enzymatic hydrolysis and increased the proportion of undigested starch, thereby decreasing the bioavailability of starch [17].

*Carya cathayensis* Sarg. (CCS, Chinese hickory) is a species within the Juglandaceae family. It is a deciduous tree characterized by its pinnately compound leaves and is predominantly cultivated in Asia. The kernel of CCS is rich in fat, protein, and sugars and contains functional phytochemicals, such as polyphenols, sterols, and tocopherols [18,19]. Thus, the kernel of CCS is considered a healthy food and is used as a raw ingredient for food production.

The CCS peel (CCSP, also known as the green husk) cracks and is removed during kernel processing and is thus considered the primary byproduct of the kernel. The CCSP decays slowly due to its content of substances such as alkaloids, which strongly inhibit microorganisms. Therefore, the discarding of a large amount of CCSP, when scoured by rain, can cause water quality deterioration and environmental pollution [20]. Strategies should thus be developed to turn CCSP waste into valuable resources. For hundreds of years, CCSP has been used in traditional Chinese medicine to treat ulcers, skin diseases, asthma, analgesia, and other ailments [21]. This therapeutic use is believed to be linked to its rich content of bioactive compounds, encompassing polyphenols, sterols, quinones, and polysaccharides [22]. As reported previously, crude extract from CCSP showed α-amylase inhibitory activity, emphasizing the importance of this plant as a source of health-promoting food [23]. However, to the best of our knowledge, the active ingredients in CCSP responsible for α-amylase inhibitory activity are not yet clear.

In the present work, the active compound with strong α-amylase inhibition activity from CCSP was discovered by bioassay-guided isolation. Moreover, the inhibitory characterization of the identified compound against α-amylase was evaluated.

## 2. Material and Method

### 2.1. Material and Chemical Reagents

The CCSP was collected from the local farmer market of Hangzhou, Zhejiang Province, China. AB-8 macroporous adsorption resin was purchased from Sunresin New Materials Co., Ltd. (Xi’an, China). Sephadex LH-20 gel was obtained from Cytiva Co., Ltd. (Shanghai, China). Soluble starch was provided by Yongda Chemical Co., Ltd. (Tianjin, China). DNS (3,5-dinitrosalicylic acid) reagent was supplied by Codow Co., Ltd. (Guangzhou, China). Acarbose, hog pancreas α-amylase, C_18_-reversed-phase silica gel, and CD_3_OD were purchased from Sigma–Aldrich Co., Ltd. (Shanghai, China). All other reagents in this study were purchased from Aladdin Reagent Co., Ltd. (Shanghai, China). Ultra-pure water was used throughout the study.

### 2.2. Preparation of Extracts from CCSP

The extraction of extracts from *Carya cathayensis* Sarg. peel (CCSP) was conducted based on a previously reported method, with certain modifications [17]. Initially, 5 kg of CCSP was thoroughly washed using tap water and subsequently dried in an oven set at 45 °C for a duration of 24 h. Post drying, the peel was ground into a fine powder using an ultrafine grinder (model DFY-500, Wenling Linda Instrument Co., Ltd., Taizhou, China), ensuring the particle size passed through a 40-mesh sieve. This powdered CCSP was then soaked in a 75% (*v*/*v*) ethanol aqueous solution, maintaining a ratio of 1:15 (*w*/*v*). The mixture underwent intermittent stirring for a period of 24 h, followed by centrifugation at 7500× *g* for 15 min. The resulting supernatant was concentrated under vacuum using a rotary evaporator (model RE-2000A, Shanghai Yarong Co., Ltd., Shanghai, China). Finally, the concentrated extract was freeze-dried, preparing for subsequent analytical procedures.

### 2.3. α-Amylase Inhibition Activity Assay

The inhibition assay was performed as described before, with some modifications [24]. Briefly, 100 μL of α-amylase (1.25 U/mL) was mixed with 100 μL of different concentrations of test samples. Then, 200 μL of starch (0.5%, *w*/*v*) dissolved in phosphate buffer was added to the mixture and incubated at 37 °C for 8 min. To terminate the enzymatic reaction, 100 μL of DNS color reagent was added. After incubation at 100 °C for 10 min, the mixture was cooled to room temperature. Finally, the mixture was diluted with 1.5 mL of water, and the absorbance at 540 nm was recorded by an UV spectrophotometer (UV-1100, Mapada Instruments Co., Ltd., Shanghai, China). The assays of the control (the inhibitor solution was replaced by the carrier solvent), the sample background (the enzyme solution was replaced by PBS buffer), and the control background (the inhibitor solution and the enzyme solution were replaced by their carrier solvents) were performed by the same method as mentioned above. Each experiment was performed in triplicate. Acarbose is a specific inhibitor of pancreatic α-amylase enzyme and acted as the positive control. The inhibition activity was calculated using the following formula:

(1) $$Inhibition\;activity(\%)=(1-\frac{A_{S}-A_{SB}}{A_{C}-A_{CB}})\times 100\%$$

where A_S_, A_SB_, A_C,_ and A_CB_ are the absorbances of the sample, sample background, control and control background, respectively.

### 2.4. Isolation of α-Amylase Inhibitor

The bioassay-guidance isolation was performed using a method previously reported with some modifications [14,25]. The lyophilized crude extract powder (25 g) was dissolved in 350 mL of water and applied to an AB-8 macroporous adsorption resin column (600 mm × 40 mm) pre-equilibrated with water. Following this, the column was purged with 2 bed volumes (BV) of water to eliminate the unadhered compounds prior to successive elution. Subsequently, the column underwent stepwise desorption using 2 BV of aqueous ethanol solution, with ethanol concentrations of 15%, 30%, 45%, 60%, and 80% (*v*/*v*), respectively. All five collected fractions (F1-F5) were vacuum-concentrated to dryness and lyophilized for the bioactive assay. Then, the fraction (F2) was applied onto a Sephadex LH-20 column (300 mm × 35 mm) pre-equilibrated with water using a gradient elution of 2 BV of 5, 10, 15, 20, and 25% ethanol aqueous solution (*v*/*v*) to give five subfractions (F2-1–F2-5). Subfraction F2-2, guided by HPLC profiles, was further purified using a C_18_-reversed-phase silica gel column (175 mm × 18 mm). Initial elution was performed with 2% methanol and 0.5% formic acid for the first 5 min, followed by a gradient increase to 15% methanol from 5 to 20 min. After four rounds of elution purification, four eluates were obtained.

### 2.5. HPLC Analysis

HPLC analysis was conducted using an Agilent 1260 system equipped with a UV detector (Agilent Technologies Co., Ltd., Palo Alto, CA, USA). For preparation, the lyophilized sample powder was dissolved in water and then filtered through a 0.22 μm syringe filter. The filtered sample solution was introduced into a Welch Ultimate LP-C_18_ column (250 mm × 4.6 mm, 5 μm), which was maintained at a constant temperature of 25 °C. The flow rate was set at 1 mL/min, and the absorbance of the eluent was monitored at 280 nm. The mobile phases used for the separation included water/formic acid (99:1, *v*/*v*, as solvent A) and acetonitrile (as solvent B). Initially, the column was equilibrated with 5% solvent B. The elution of sample compounds was conducted in a stepwise manner, with the following gradient profile: from 0 to 20 min, the concentration of solvent B was increased from 5% to 35%; from 20 to 25 min, it was ramped up from 35% to 100%; and from 25 to 30 min, the system was held at 100% solvent B. 

### 2.6. Nuclear Magnetic Resonance (NMR) and Q-Exactive Orbitrap Mass Spectrometry Assays

The Nuclear Magnetic Resonance (NMR) and Q-Exactive Orbitrap Mass Spectrometry assays were employed to analyze the purified compound, adhering to established protocols with modifications for enhanced accuracy and sensitivity [11,26]. NMR spectra (^1^H and ^13^C) were recorded on a Bruker Avance III 400 MHz spectrometer (Bruker BioSpin, Karlsruhe, Germany). The compound was dissolved with CD_3_OD for measurement. Mass spectrometry was determined on a Thermo Scientific Q Exactive™ quadrupole-Orbitrap mass spectrometer (San Jose, CA, USA). The MS analysis conditions were as follows: scan type was Full-ddMS2; polarity was positive/negative modes; sheath gas flow rate was 45 arbitrary units; aux gas flow rate was 10 arbitrary units; spray voltage was set at 3.5 kV; capillary temperature was 275 °C; and gas heater temperature was set at 400 °C.

### 2.7. Inhibitory Kinetics Analysis

The inhibitory kinetic characteristics of 5-CQA against α-amylase were assessed based on a modified method from a previous study [15]. Calculations were performed using the Michaelis−Menten equation to determine the Michaelis constant (K_m_) and maximum reaction velocity (V_max_) at various concentrations of 5-CQA solution. To calculate the dissociation constants for competitive inhibition (K_ic_) and noncompetitive inhibition (K_in_), the Michaelis−Menten equations were presented as follows [27]:

(2) $$Competitive\;inhibition\;type:V=\frac{V_{m}[S]}{K_{m}(1+\frac{\left[I\right]}{K_{ic}})+[S]}$$

(3) $$Noncompetitive\;inhibition\;type:V=\frac{V_{m}[S]}{\left(1+\frac{\left[I\right]}{K_{in}})(K_{m}+[S]\right)}$$

where V is the initial velocity, V_m_ is the maximum velocity, K_m_ is the Michaelis constant for the substrate (starch), [I] is the concentration of the inhibitor, [S] is the concentration of substrate (starch), K_ic_ is the constant for competitive inhibition, and K_in_ is the constant for noncompetitive inhibition.

To generate the Lineweaver–Burk plot, 1/V was plotted against 1/[S], with varying inhibitor concentrations. Additionally, plots of 1/[V] and [S]/V were constructed to conduct the Dixon plot and Eisenthal−Cornish−Bowden plot, correspondingly, at various substance concentrations.

### 2.8. Statistical Analysis

The results were presented as mean ± standard deviation (SD) for triplicate. Data were analyzed using SPSS version 26.0 software (Chicago, IL, USA), employing one-way ANOVA followed by Duncan’s test to evaluate significant differences, with a significance level set at *p* < 0.05. Graphical representations of the data were generated using Origin 2021.

## 3. Results and Discussion

### 3.1. Purification of α-Amylase Inhibitor from CCSP

**Figure 1:**
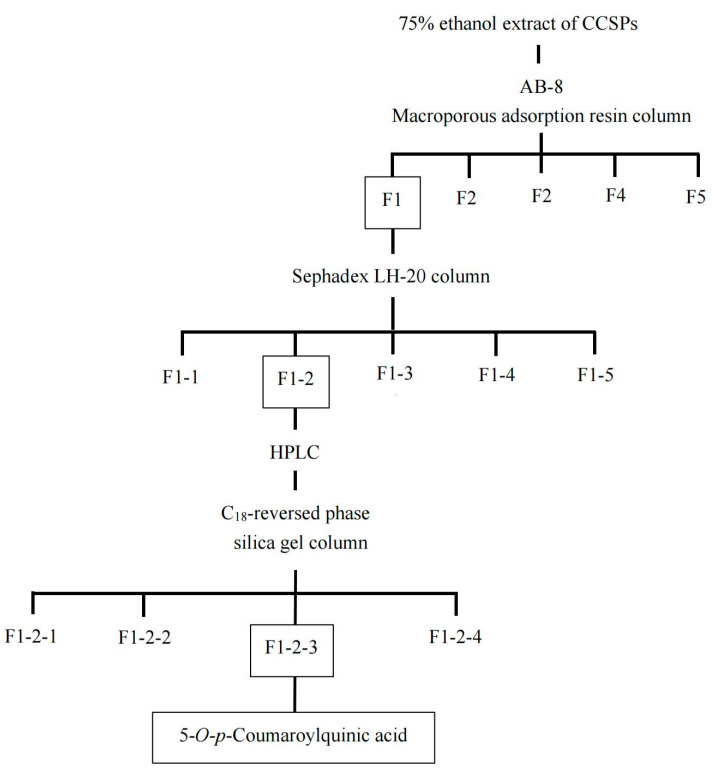
Schematic diagram of the procedure for isolation of an α-amylase inhibitor, 5-*O-p*-coumaroylquinic acid (5-CQA), from *Carya cathayensis* Sarg. Peel (CCSP). Boxed fractions showed the strongest α-amylase inhibitory activity.

**Figure 2:**
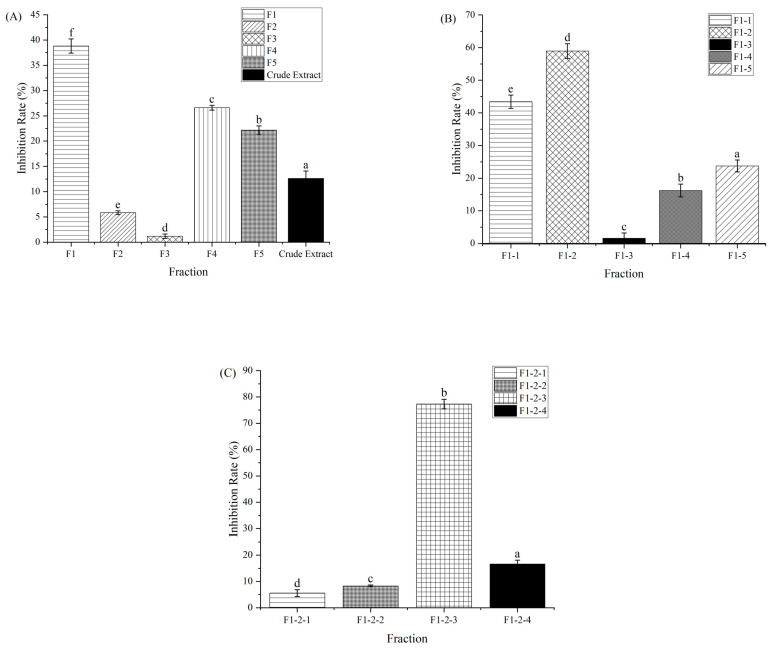
α-Amylase inhibitory activity of fractions collected from the columns. (**A**) Crude extract and F1–F5 collected from the AB-8 macroporous adsorption resin column (at a concentration of 1000 µg/mL). (**B**) F1-1–F1-5 collected from the Sephadex LH-20 column (at a concentration of 600 µg/mL). (**C**) F1-2-1–F1-2-4 collected from the C_18_ reversed-phase silica gel column (at a concentration of 200 µg/mL). Different letters above the columns in the same figure indicate a significant difference at *p* < 0.05.

The purification procedure to obtain the component with α-amylase inhibitory activity is shown in Figure 1. Macroporous adsorption resin has been extensively used to separate bioactive components from plant [11]. The crude extract of CCSP was loaded on an AB-8 macroporous adsorption resin column, and the eluent was partitioned into five fractions (F1–F5). Compared with other four fractions, F1 showed the highest activity (38.81%) at 1000 µg/mL, as shown in Figure 2A. In chromatography utilizing macroporous adsorption resin, an inverse relationship was observed between the polarity of the eluted component and the concentration of the alcohol solution when alcohol–water mixtures are employed as eluents [25,28,29]. In this study, the active fraction (F1) was eluted using a 15% alcohol solution, suggesting that strongly hydrophilic compounds contributed to the α-amylase inhibitory activity. Hence, F1 was subjected to further separation. 

**Figure 3:**
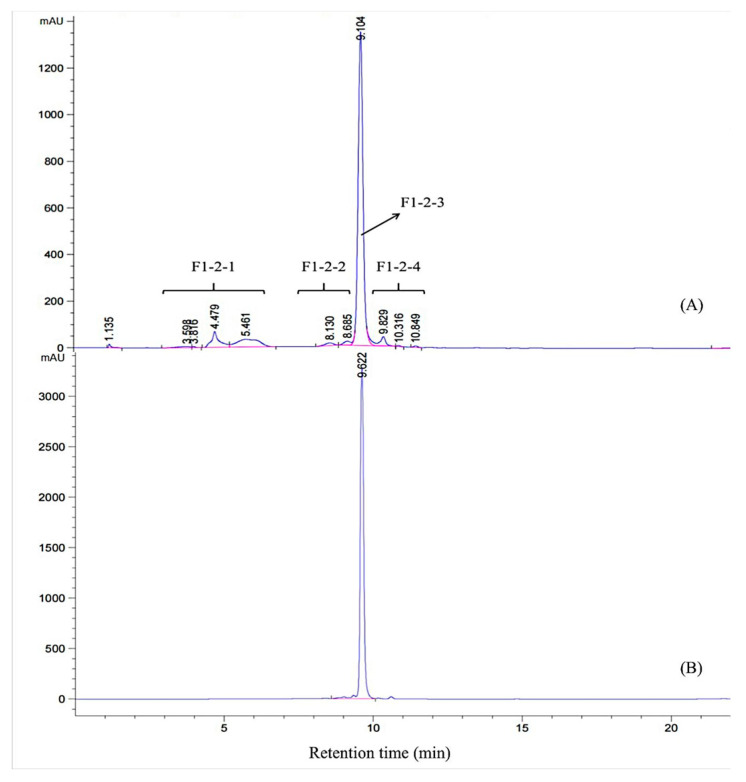
HPLC chromatogram of the F1-2-2 fraction (**A**) and the purified compound from F1-2-2 (**B**).

F1 was eluted on a Sephadex LH-20 column, and five fractions (F1-1–F1-5) were collected for activity assay. Fraction F1-2 exhibited the highest inhibitory activity (58.94%) at 600 µg/mL compared with the other subfractions (Figure 2B). The HPLC profile of F1-2 is shown in Figure 3A. F1-2 was composed primarily of four subfractions (F1-2-1, F1-2-2, F1-2-3 and F1-2-4), corresponding to retention time ranges of 2.5–7.3, 7.5–8.7, 8.8–9.7, and 9.8–11.0 min, respectively. In order to scale up the number of compounds, a C_18_ reversed-phase silica gel column was employed to purify the four subfractions from F1-2. Among the four subfractions of F1-2, F1-2-3 demonstrated the highest potential, exhibiting inhibitory activity of 77.30% at a concentration of 200 µg/mL (Figure 2C). HPLC chromatograms of F-1-2-3 are shown in Figure 3B. Thus, F-1-2-3 was identified as an α-amylase inhibitor and was subjected to structural measurement.

### 3.2. Identification of Active Compound

**Figure 4:**
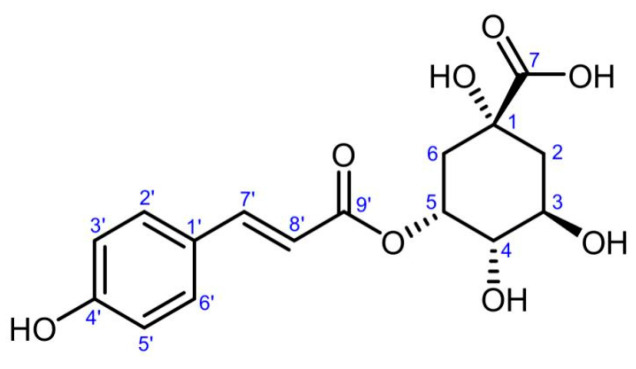
The chemical structure of 5-*O-p*-coumaroylquinic acid.

The active compound obtained was a red amorphous powder. Mass spectra are shown in Figure S1. The molecular formula was determined to be C_16_H_18_O_8_ by Q-Exactive Orbitrap Mass Spectrometry (337.0930 [M−H]^−^, 339.1073 [M+H]^+^ and 361.0891 [M-Na]^+^). The positive fragment ion with an m/z value of 147.0440 [M-quinic acid+H]^+^ indicated the presence of a quinic acid moiety in the compound. ^1^H-NMR (CD_3_OD, 400 MHz) (Figure S2A): δH 7.64 (1H, d, J = 15.8 Hz, H-7′), 7.44 (2H, d, J = 8.2 Hz, H-2′, H-6), 6.80 (2H, d, J = 8.2 Hz, H-3′, H-5′), 6.36 (1H, d, J = 15.8 Hz, H-8′), 5.36 (1H, br s, H-5), 4.16 (1H, ddd, J = 10.0, 8.5, 3.7 Hz, H-3), 3.64 (1H, dd, J = 8.5, 3.0 Hz, H-4), 2.07–2.24 (3H, m, H-2α, H-6), 1.95 (1H, dd, J = 13.1, 10.0 Hz, H-2β). ^13^C-NMR (CD_3_OD, 100 MHz) (Figure S2B): δC 178.3 (s, C-7), 169.0 (s, C-9′), 161.0 (s, C-4′), 146.4 (d, C-7′), 131.1 (d, C-2′, C-6′), 127.4 (s, C-1′), 116.8 (d, C-3′, C-5′), 115.9 (d, C-8′), 75.4 (s, C-1), 74.8 (d, C-4), 73.0 (d, C-5), 68.2 (d, C-3), 41.5 (t, C-2), 36.7 (t, C-6). The NMR data were closely consistent with those previously reported [30,31]. Thus, the compound was identified as 5-*O-p*-coumaroylquinic acid (5-CQA), and the chemical structure is shown in Figure 4. To our knowledge, this compound was first reported in *Carya cathayensis* Sarg.

### 3.3. Inhibitory Activity of 5-CQA against α-Amylase

**Figure 5:**
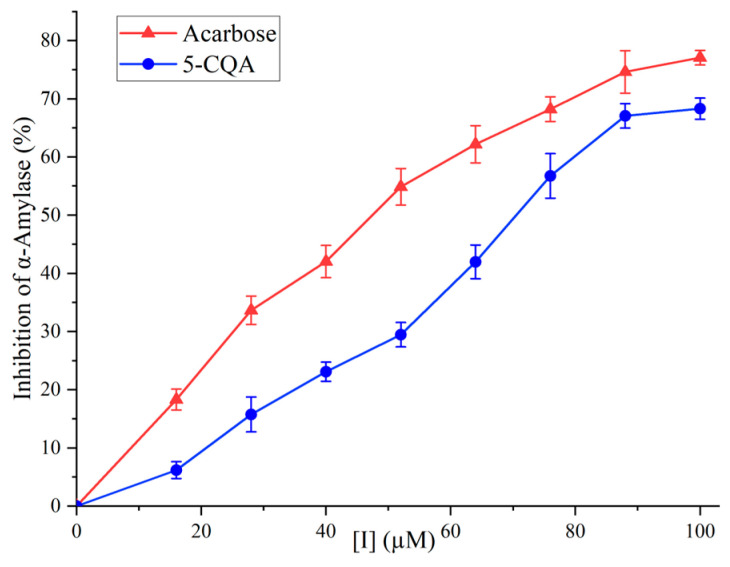
The inhibitory activities of 5-CQA and acarbose on α-amylase.

The inhibitory activity of 5-CQA against α-amylase is shown in Figure 5. In the concentration range of 0 to 100 µg/mL, 5-CQA exhibited inhibition in a dose-dependent manner. At 100 µM, the inhibitory activity of 5-CQA reached 68.30%. The IC50 values for 5-CQA and acarbose were 69.39 µM and 45.39 µM, respectively, demonstrating that 5-CQA was an effective inhibitor against α-amylase. 

Though 5-CQA was less potent than acarbose, it demonstrated a stronger inhibitory effect compared to certain previously studied phenolic acids, such as gallic acid (IC50 of 1.25 mM), vanillic acid (IC50 of 27.89 mM), and syringic acid (IC50 of 44.81 mM) [32,33]. A significant advantage of 5-CQA, similar to other natural plant-derived inhibitors, lied in its enhanced biocompatibility with human physiological systems. This compatibility generally translates into a lower probability of adverse side effects, a notable benefit when contrasted with many synthetic inhibitors, which may present higher toxicity [34]. Furthermore, natural inhibitors like 5-CQA extend health benefits beyond mere enzyme inhibition. The utilization of plant-derived inhibitors such as 5-CQA offered not only the primary function of α-amylase inhibition but also additional advantages, including antioxidant properties, often absent in synthetic hypoglycemic drugs [35]. Another aspect to consider is the cost-effectiveness of natural inhibitors like 5-CQA. Depending on the source and extraction methods, these inhibitors might be more economical, particularly in regions with abundant source plants [36]. Together, these characteristics emphasize the potential of 5-CQA from natural sources as a valuable option in both therapeutic and economic terms. 

In the process of dietary carbohydrate digestion, α-amylase plays a pivotal role by catalyzing the breakdown of starch into oligosaccharides. These oligosaccharides are then further hydrolyzed into glucose by α-glucosidase, facilitating rapid absorption by the body [37]. The interaction of 5-CQA with α-amylase leads to an inhibition of the enzyme’s activity, thereby slowing down the rate of starch hydrolysis in ingested food. This inhibition results in a consequent reduction in the glycemic index of the consumed carbohydrates, contributing to a moderated rise in blood glucose levels post-meal [38]. Inhibition of α-amylase indicates that 5-CQA has the ability to retard dietary starch liberation and reduce its absorption, resulting in controlled postprandial hyperglycemia. Similar results were also described in other phenolic acid compounds, such as chlorogenic acid [39] and ferulic acid [40], which effectively inhibited α-amylase.

Key amino acid residues within the α-amylase enzyme, namely Glu233, Asp197, and Asp300, play a crucial role in starch hydrolysis. These residues also form the binding sites for enzyme inhibitors at the active site [41]. The interaction of inhibitors with α-amylase, either directly or indirectly, induces conformational changes in the enzyme, potentially leading to the deactivation of the active sites. These interactions encompass a spectrum of molecular forces, including hydrogen bonding, van der Waals forces, electrostatic interactions, and hydrophobic contacts [42]. Plant-derived phenolic acids, such as gallic acid, vanillic acid, and cinnamic acid, are well documented for their pronounced inhibitory effects on α-amylase [41,43]. The inhibitory activity of phenolic acids is largely dependent on their specific chemical structures. It has been demonstrated that the hydrogen bonding interaction between phenolic acids and α-amylase plays a crucial role in this inhibitory mechanism [44,45]. Recently, an investigation employing saturation transfer difference NMR revealed the hydrophobic interaction between the aromatic ring of phenolic acid and α-amylase, thereby inhibiting the enzyme [46]. It is hypothesized that the hydrogen bonding and hydrophobic interactions are key contributors to the inhibitory activity of phenolic acids. These interactions with α-amylase potentially induce conformational alterations in the enzyme’s protein structure.

### 3.4. Inhibitory Kinetics of 5-CQA against α-Amylase

**Figure 6:**
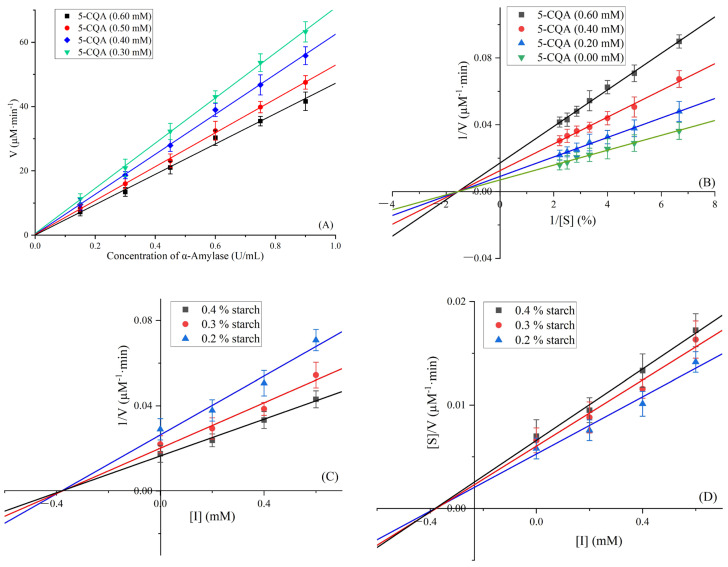
The plot of the initial velocity (V) against the concentration of α-amylase (**A**), Lineweaver–Burk plots (**B**), Dixon plots (**C**), and Eisenthal–Cornish-Bowden plots (**D**) of 5-CQA against α-amylase.

Figure 6A displays the plot of the initial velocity (V) against the concentration of α-amylase, while considering various concentrations of 5-CQA. As the inhibitor concentration increased, all the linear trends intersected at the origin of coordinate, and the slope gradually diminished. The evidence suggests that 5-CQA could act as a reversible inhibitor, diminishing the catalytic activity of α-amylase. 

The Lineweaver–Burk plots of 5-CQA against α-amylase are presented in Figure 6B. These plots converged at a single point on the X-axis. Using Equation (2), the values of K_m_ and V_max_ were calculated through linear fitting, as detailed in Table 1. As the concentration of 5-CQA increased, the K_m_ values remained consistent at approximately 0.64 µM, while the V_max_ values decreased from 144.30 to 58.51 µM·min^−1^. These findings suggest that 5-CQA behaves as a noncompetitive inhibitor against α-amylase.

Dixon and Eisenthal–Cornish-Bowden plots are shown in Figure 6C,D, respectively. All lines intersected at a single point on the X-axis in both plots, further supporting that 5-CQA was a noncompetitive inhibitor. The results indicate that 5-CQA acted as a noncompetitive inhibitor of α-amylase by binding to the inactive sites on α-amylase and the starch–α-amylase complex. Values of K_ic_ and K_in_ were calculated according to Equations (2) and (3) and shown in Table 1. The K_ic_ value and K_in_ value were approximately the same (0.38 µM), indicating 5-CQA bound to the α-amylase with the same affinity as the starch–α-amylase complex [47]. Similar findings were corroborated by previous studies [37,48], affirming that phenolic acids functioned as noncompetitive inhibitors of α-amylase from hog pancreas.

The commercially available α-amylase inhibitor acarbose is a competitive inhibitor. This means it competes with starch, the natural substrate of α-amylase, for binding to the enzyme. By doing so, it effectively reduces the rate at which starch is broken down into glucose, leading to a slower and reduced absorption of glucose in the intestines [49]. Since acarbose works in the gastrointestinal tract and inhibits carbohydrate digestion, it can lead to gastrointestinal side effects like flatulence, bloating, and diarrhea. These effects are due to the fermentation of undigested carbohydrates in the large intestine [50]. 5-CQA, in contrast, is a noncompetitive inhibitor of α-amylase. It binds to a site on the enzyme different from the active site (allosteric site), altering the enzyme’s shape or function. This alteration reduces the enzyme’s activity without direct competition with the substrate (starch) [51]. Although acarbose and 5-CQA both effectively reduce postprandial blood glucose levels, the efficacy of acarbose, being a competitive inhibitor, diminishes in environments with elevated substrate concentrations [52,53]. In contrast, the effectiveness of 5-CQA, functioning as a noncompetitive inhibitor, remains unaffected by the concentration of carbohydrate substrates, thereby indicating a potential risk for hypoglycemia [52,54]. Acarbose is primarily used as a therapeutic agent for diabetes management, whereas 5-CQA, found in dietary sources, might be viewed more as a preventive or complementary intervention with additional health benefits. 

## 4. Conclusions

In summary, this study successfully isolated and identified 5-*O-p*-coumaroylquinic acid (5-CQA) as an α-amylase inhibitor from CCSP, demonstrating its inhibitory activity in a reversible and noncompetitive manner. These findings indicate that 5-CQA is a novel and promising candidate for α-amylase inhibition and has potential as a functional food ingredient for managing postprandial hyperglycemia. To further explore the efficacy of 5-CQA within a complex physiological environment, future in vivo studies, utilizing diabetic mouse models, are essential.

## Figures and Tables

**Table 1:** Kinetic Parameters of α-Amylase treated with 5-*O-p*-coumaroylquinic acid (5-CQA) *.

Concentration of 5-CQA (mM)	Michaelis Constant (K_m_) (µM)	Maximum Velocity (V_max_) (µM·min^−1^)	Constant for Competitive Inhibition (K_ic_) (µM)	Constant for Noncompetitive Inhibition (K_in_) (µM)
0	0.64	144.30	0.38 ± 0.0022	0.38 ± 0.0024
0.20	0.65	110.62		
0.40	0.64	79.94		
0.60	0.64	58.51		

* Mean ± SD.

## Data Availability

Data are contained within the article.

## Supplementary Materials

The following supporting information can be downloaded at: https://www.mdpi.com/article/10.3390/foods12244425/s1, Figure S1: Mass spectra of the active compound in positive and negative ionization modes, Figure S2: NMR spectra of the active compound. (A) ^1^H-NMR spectra. (B) ^13^C-NMR spectra.
