# Supplementary material for: Screening and Characterization of an α-Amylase Inhibitor from Carya cathayensis Sarg. Peel
Source: Foods. 2023 Dec 10;12(24):4425. doi: 10.3390/foods12244425 (PMC10742785; doi:10.3390/foods12244425)
Supplement: Supplementary file 1 [file foods-12-04425-s001.zip › Figure_S1.pdf]

Figure S1.

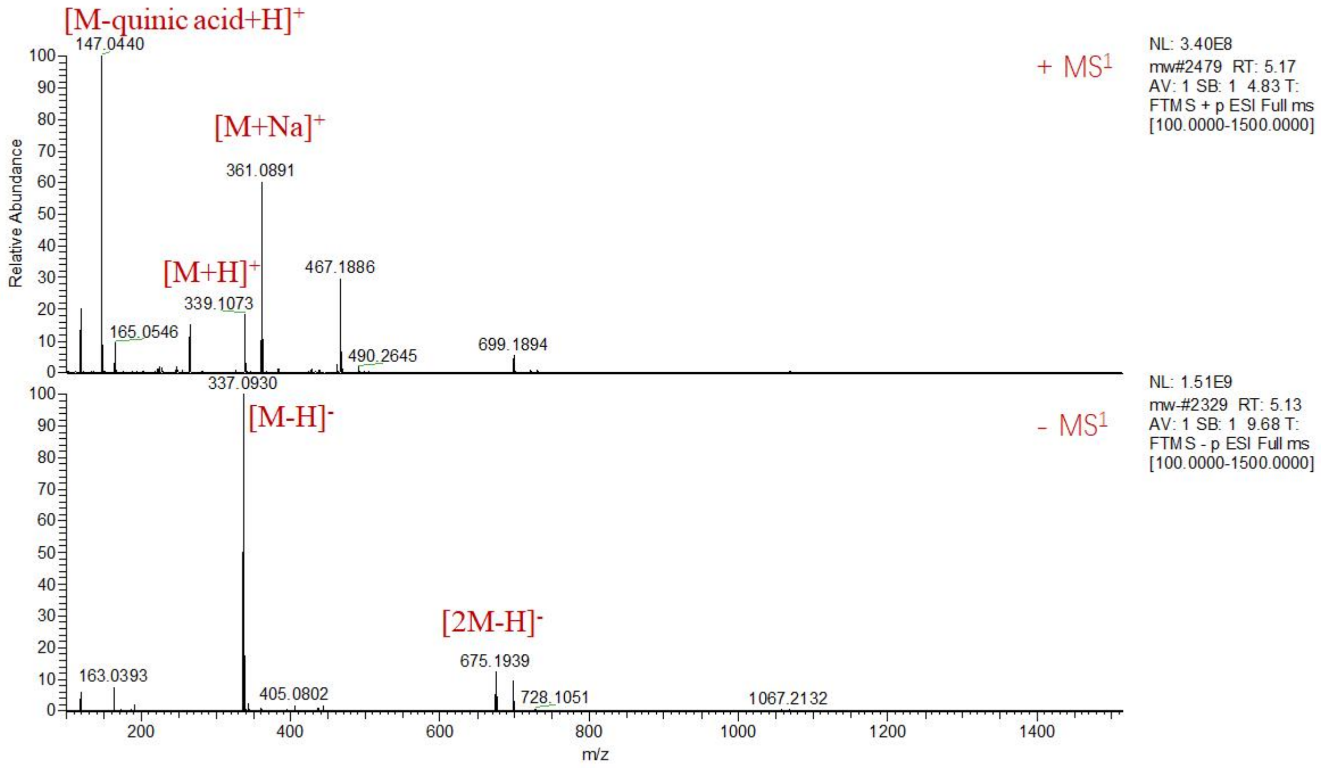

Figure S1: Mass spectra of the active compound in positive and negative ionization modes.
