# Supplementary figures and images for: Screening and Characterization of an α-Amylase Inhibitor from Carya cathayensis Sarg. Peel
Source: Foods. 2023 Dec 10;12(24):4425. doi: 10.3390/foods12244425 (PMC10742785; doi:10.3390/foods12244425)

Fig. S2.

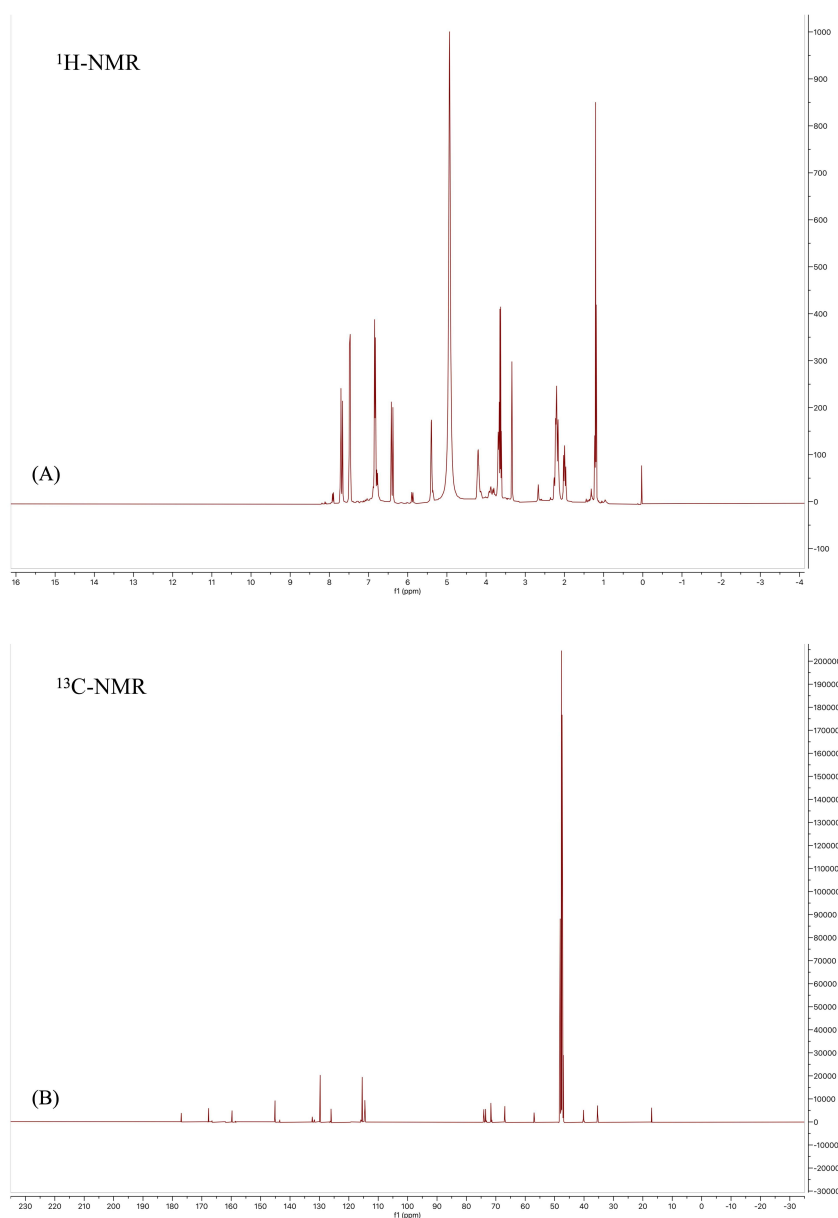

Figure S2: NMR spectra of the active compound. (A)  $^1\text{H}$ -NMR spectra. (B)  $^{13}\text{C}$ -NMR spectra.

Supplement: Supplementary file 1 [file foods-12-04425-s001.zip › Figure_S2.pdf]
